# Pregnancy in women with autosomal recessive Alport syndrome caused by novel compound heterozygous mutations of *COL4A3* gene: Two cases reports

**DOI:** 10.1097/MD.0000000000036057

**Published:** 2023-11-17

**Authors:** Xiaoli Gao, Meilu Li, Kan Wang, Zengyan Li, Cha Han

**Affiliations:** a Department of Gynecology and Obstetrics, Tianjin Medical University General Hospital, Tianjin, China; b Tianjin Key Laboratory of Female Reproductive Health and Eugenics, Tianjin Medical University General Hospital, Tianjin, China.

**Keywords:** ARAS, *COL4A3*, compound heterozygous mutations, pregnancy, progressive proteinuria

## Abstract

**Rationale:**

Autosomal recessive Alport syndrome (ARAS) is an hereditary heterogeneous disease that poses a serious risk to pregnant women.

**Patient concerns:**

We reported 2 cases of pregnancy with progressive proteinuria. The case 1 was a 21-year-old woman with 24-h proteinuria increased from 2.03 to 11.72 g at 13 to 35 weeks of gestation, and the case 2 was a 28-year-old woman with 24-h proteinuria increased from 2.10 to 9.32 g at 8 to 36 weeks of gestation. In advanced stage of pregnancy, the fetal development was smaller than the gestational age.

**Diagnoses:**

Sanger sequencing showed that novel compound heterozygous mutations [c.1315 G>T (p.G439C) and c.4847 G>A (p.C1616Y)] of the collagen type IV alpha 3 chain (*COL4A3*) gene were found in the 2 cases. Renal puncture pathology confirmed the diagnosis of ARAS.

**Interventions:**

The 2 cases were treated with albumin, compounded amino acids, calcium, vitamin D, and low molecular weight heparin in addition to conventional treatment during pregnancy. Pregnancy was terminated by cesarean section at 36 to 37 weeks of gestation. After delivery, the patients were treated with Losartan for anti-proteinuric therapy for 1 year.

**Outcomes:**

The neonatal weights and Apgar scores were normal. The patients recovered well and 24-h proteinuria decreased to pre-pregnancy level.

**Lessons:**

When pregnant women present with a persistent increasing proteinuria, ARAS needs to be considered. Sanger sequencing is useful to assist in the diagnosis of ARAS. Multidisciplinary treatments from nephrologists and gynecologists are needed to ensure the safety of pregnancy and the fetus.

## 1. Introduction

Alport syndrome (AS) is an hereditary heterogeneous disease, which is mainly characterized by hematuria, proteinuria and progressive kidney dysfunction, with sensorineural deafness and eye abnormalities in some patients.^[1]^ Epidemiological studies showed that the prevalence of AS is estimated at 1:50,000 of the population worldwide.^[2]^ The AS is caused by mutations in the genes encoding the collagen type IV alpha 3, 4, 5 chains (*COL4A3, COL4A4*, and *COL4A5*).^[3,4]^ To date, at least 1422 mutations causing AS have been reported in previous literatures.^[5]^ Approximately 85% of cases are X-linked AS (XLAS), caused by mutations in the *COL4A5* gene. About 5% of cases are autosomal recessive AS (ARAS) and rare (<5%) are autosomal dominant AS (ADAS), caused by mutations in the *COL4A3* and *COL4A4* genes.

Currently, due to the unspecific clinical manifestations and the progression towards a renal phenotype with glomerulonephritis, ARAS remains a highly misdiagnosed entity. Pregnancy in women with ARAS have an increased risk of impaired kidney function and preeclampsia.^[6]^ Diagnosis is conventionally made pathologically, but recent advances in comprehensive genetic analysis have enabled genetic testing to be performed for the diagnosis of ARAS in first-line diagnosis.^[7]^ Improved mutation detection has led to the identification of several pathogenic variants in ARAS.^[8]^ It illustrates that ARAS phenotypes vary widely as the result of the specific genetic variants present. Here, we described the processes of gestation, pregnancy outcome, and postpartum follow up for 2 cases of pregnancy in women with ARAS caused by novel compound heterozygous mutations in the *COL4A3* gene.

## 2. Case presentation

### 2.1. Case 1

The case 1 was a 21-year-old pregnant woman, gravid 1 Para 0, with height at 160 cm and weight at 45 kg. The patient was first found to have abnormal proteinuria (+++) and abnormal urine occult blood (++) at the 13 weeks of gestation. Hematological test showed mild anemia (hemoglobin: 107 g/L), and biochemical test showed hypoproteinemia (total protein: 53.8 g/L, albumin: 29.2 g/L). At 13 to 26 weeks of gestation, 24-h proteinuria fluctuated at 2.03 to 3.45 g and albumin fluctuated at 24 to 29 g/L. At 27 weeks of gestation, 24-h proteinuria at 5.05 g, 4-dimensional ultrasound showed the fetal development was smaller than gestational week for 1 week. The edema of the lower extremities was positive (+). The patient was hospitalized for conventional treatment and was given intravenous infusion of albumin (10 g) every 2 weeks, oral compound amino acids, calcium and vitamin D, and subcutaneous injection of 4000 IU low molecular weight heparin (LMWH) once every other day for anticoagulation therapy. At 35 weeks of gestation, 24-h proteinuria at 11.72 g, the fetal development was smaller than the gestational week for 2 weeks. The pregnancy was terminated by cesarean operation at 36 weeks of gestation, and a live boy was delivered with weight at 2400 g and Apgar score at 9’-10’-10’.

**Figure 1.**
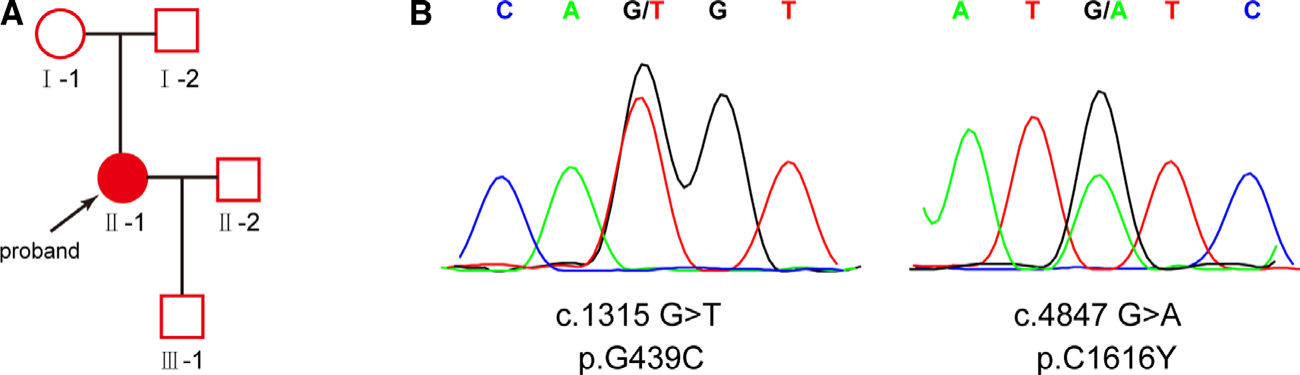
Identification of compound heterozygous mutations of *COL4A3* gene in the case 1 with ARAS. (A) Pedigree and genetic typing of the case 1 with ARAS. Filled and open signs indicate affected and unaffected individuals, respectively. The proband was marked with a black arrow. (B) Sanger sequencing confirmed the compound heterozygous mutations [c.1315 G>T (p.G439C) and c.4847 G>A (p.C1616Y)] of *COL4A3* gene in the case 1.

Three months after delivery, 24-h proteinuria at 13.03 g. After 1 year of postpartum follow-up, high proteinuria persisted, 24-h proteinuria fluctuated at 2.67 to 5.31 g, the patient showed a high-frequency sensorineural hearing loss. Based on the above clinical phenotype and laboratory results, and neither of the patient’s parents had the phenotype, which is highly suggestive for ARAS. Therefore, peripheral venous blood was collected from the subject, and genomic DNA was extracted with a DNA extraction kit (TaKaRa, Beijing, China). The all exons of human *COL4A3* and *COL4A4* genes were detected using Sanger sequencing. The novel compound heterozygous mutations [c.1315 G>T (p.G439C) and c.4847 G>A (p.C1616Y)] of *COL4A3* gene were found in the patient (Fig. 1). Further pathological examination of renal puncture showed mild hyperplasia of mesangial cells and stroma, uneven thickness of basement membrane with diffuse stratified changes in the dense layer, extensive fusion of epithelial foot processes, and vacuolar degeneration of renal tubular, which confirmed the diagnosis of ARAS. Subsequently, the patient was treated with Losartan for anti-proteinuric therapy for 1 year, 24-h proteinuria decreased to pre-pregnancy level. Her son had developing normally and normal proteinuria.

### 2.2. Case 2

The case 2 was a 28-year-old pregnant woman, gravid 2 Para 0, with height at 163 cm and weight at 53 kg. The patient was first found to have abnormal proteinuria (++) and abnormal urine occult blood (+) at 2 years before pregnancy. At 8 to 27 weeks of gestation, 24-h proteinuria fluctuated at 2.10 to 3.94 g and albumin fluctuated at 25 to 29 g/L. The 4-dimensional ultrasound showed the fetal development was consistent with the gestational age. The edema of the lower extremities was negative (−). From 9 weeks of gestation, 4000 IU LMWH once every other day was used for anticoagulation therapy, intermittent infusion of albumin (10 g), and oral compound amino acids, calcium and vitamin D supplementation were given to prevent the occurrence of preeclampsia. At 29 weeks of gestation, 24-h proteinuria at 6.84 g, and the fetal development was smaller than the gestational age for 1 week. At 36 week of gestation, 24-h proteinuria at 9.32 g, and the fetal development was smaller than the gestational age for 3 weeks, with biparietal diameter at 8.8 cm, abdominal circumference at 28.3 cm, amniotic fluid index at 13.1 cm, and umbilical artery blood flow S/D value at 1.99. The pregnancy was terminated by cesarean section at 37 weeks of gestation, and a live girl was delivered with weight at 2370 g and Apgar score at 9’-10’-10’.

**Figure 2.**
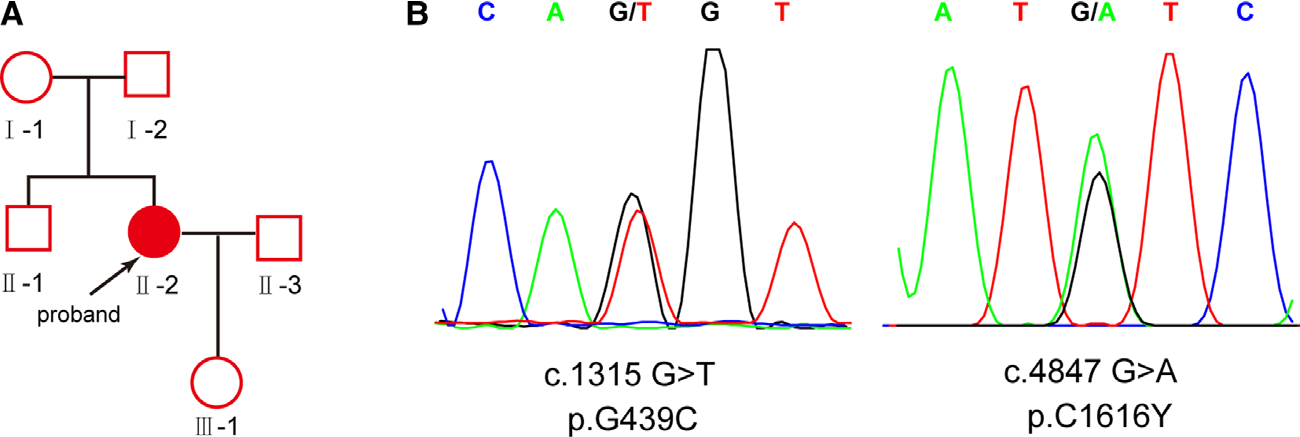
Identification of compound heterozygous mutations of *COL4A3* gene in the case 2 with ARAS. (A) Pedigree and genetic typing of the case 2 with ARAS. (B) Sanger sequencing confirmed the [c.1315 G>T (p.G439C) and c.4847 G>A (p.C1616Y)] mutations in the case 2.

One months after delivery, 24-h proteinuria at 7.26 g. After 1 year of postpartum follow-up, high proteinuria persisted, 24-h proteinuria fluctuated at 3.26 to 6.84 g. The patient had no manifest ocular or inner ear involvement. The patient denied consanguineous marriage, had no family history of hematuria, proteinuria, hearing impairment, and vision loss. Sanger sequencing revealed that patient had novel compound heterozygous mutations [c.1315 G>T (p.G439C) and c.4847 G>A (p.C1616Y)] in *COL4A3* gene (Fig. 2), most consistent with ARAS. Light microscopic examination of kidney biopsy indicated that the glomerular basement membrane was extremely irregular, and the dense layer was torn and delaminated, forming a basket-like structure, which confirmed the diagnosis of ARAS. After Losartan treatment for 1 year, the patients recovered well and 24-h proteinuria decreased to 1.32 g. Her daughter had developing normally and normal proteinuria.

## 3. Discussion

ARAS is a rare monogenic hereditary disease caused by homozygous or compound heterozygous mutations in the *COL4A3* and *COL4A4* genes.^[9]^ The prevalence of ARAS is underestimated because many cases of childhood hematuria were diagnosed as being of thin basement membrane or benign familial hematuria. To date, several novel mutations in ARAS have been reported in previous literatures.^[8,10]^ For example, Webb et al, found that a homozygous c.40_63del mutation of *COL4A3* causes ARAS in the Ashkenazi Jewish population using linkage analysis and next generation sequencing.^[11]^ Uzak et al, demonstrated that a novel missense mutation (c.2T>C) in exon 1 of *COL4A3* gene is the cause of ARAS in a large Turkish family using Sanger sequencing.^[12]^ Jehn et al, revealed that a case of ARAS in a 28-year-old woman is caused by a novel mutation (Gly1436del) in the *COL4A4* gene.^[13]^ In addition, Liao et al, suggested that a compound heterozygous mutation [c.4758_4760delCCC (p.1586_1587del) and c.4333 + 3A>G] in the *COL4A4* gene is the causative gene for ARAS.^[14]^ Pregnancy in patients affected by ARAS should be associated with maternal and fetal risks. Unfortunately, evidence on the course of pregnancy in women with ARAS is extremely scanty. To the best of our knowledge, we first presented 2 case of ARAS caused by novel compound heterozygous mutations (c.1315 G>T and c.4847 G>A) in *COL4A3* gene, which were fulminantly exposed by pregnancy.

Human *COL4A3* (NM_000091.5), consisting of 54 exons, is located at 2q36.3 (GRCh38.p14). COL4A3 protein (NP_000082.2) belongs to type IV collagen family, consisting of 1670 amino acids, molecular mass of 161 kDa. According to the expression profile revealed by the Human Protein Atlas, COL4A3 protein is mainly expressed in the basement membranes and Bowman capsules in the kidneys, cochlea, retina, lens, cornea, skin, and smooth muscles. Previous studies have shown that *COL4A3* variants are closely linked to different kidney diseases including ARAS.^[15,16]^ AS has been considered rare because the reported prevalence of AS varies from one in 5000 to one in 53,000 individuals.^[17]^ In this study, we reported a 21-year-old woman and a 28-year-old woman of pregnancy with progressive proteinuria, which were in line with the age of onset of AS.^[18]^ Meanwhile, massive proteinuria was detected in the 2 cases during the 29 to 35 weeks of gestation, which was typical for pregnant women with AS.^[19]^ Given the clinical presentation, the 2 cases were monitored by a multidisciplinary team of nephrologists and gynecologists during the pregnancy, and were treated with albumin, compounded amino acids, calcium, vitamin D, and LMWH in addition to conventional treatment. Regarding the fetal outcome, the main risk for the fetus from ARAS is prematurity. In our report, pregnancy was terminated by cesarean section at 36 to 37 weeks of gestation, and neonatal weights and Apgar scores were normal, suggesting the pregnancy outcome was good.

**Figure 3.**
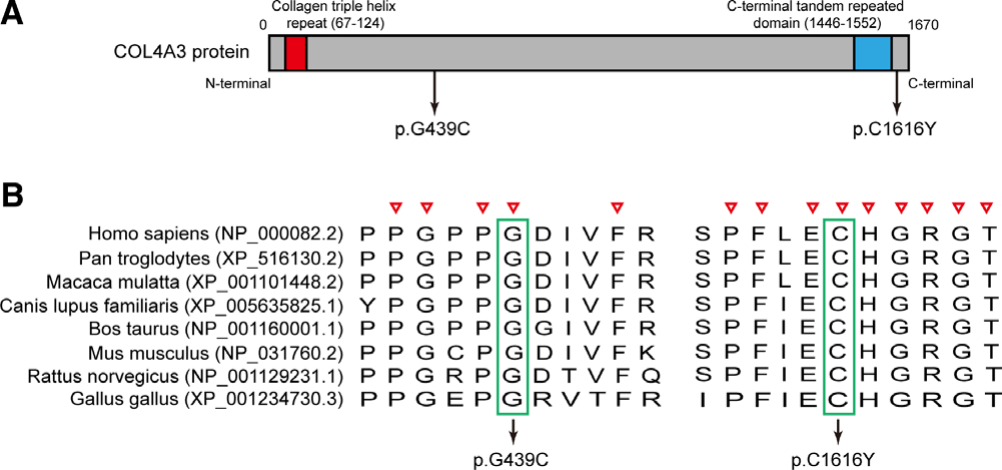
The evolutionary conservation analysis of p.G439C and p.C1616Y mutations in COL4A3 protein. (A) Schematic representation of human COL4A3 protein and the p.G439C and p.C1616Y mutations. (B) The evolutionary conservation of each amino acid altered by the 2 mutations was analyzed via multiple protein sequence alignments from *Homo sapiens, Pan troglodytes, Macaca mulatta, Canis lupus familiaris, Bos taurus, Mus musculus, Rattus norvegicus*, and *Gallus gallus*. The red triangle represented the conserved amino acids, and the mutation sites were marked with green box.

After delivery, high proteinuria persisted in the 2 cases. Progressive proteinuria with advancing gestation and postpartum are indeed a common finding that was present in all the reported cases of AS in previous literatures.^[20,21]^ The 2 cases were descended from nonconsanguineous parents, and their parents were completely asymptomatic with normal proteinuria, indicating a recessive genetic disorder. Therefore, ARAS was suspected in the 2 cases. After obtaining consent from the patients, we performed DNA sequencing of the all exons of *COL4A3* and *COL4A4* genes using Sanger sequencing. Sequencing revealed 2 novel heterozygous missense mutations [c.1315 G>T (p.G439C) and c.4847 G>A (p.C1616Y)], both in the *COL4A3* gene. When the compound heterozygous mutations occurred, the original glycine and cysteine were replaced by cysteine and tyrosine, respectively. The mutations were either rare or absent in public population genome databases. The evolutionary conservation analysis showed that amino acids at 439 and 1616 of COL4A3 protein were evolutionarily conserved (Fig. 3). The compound heterozygous mutations were predicted by multiple bioinformatics tools including SIFT (http://blocks.fhcrc.org/sift/SIFT/), MutationTaster (https://www.mutationtaster.org/), and PolyPhen-2 (http://genetics.bwh.harvard.edu/pph2/), which were deleterious to the function of COL4A3 protein (Table 1). More importantly, kidney biopsy confirmed the diagnosis of ARAS.

**Table 1:** Assessment of the compound heterozygous mutations that affected COL4A3 protein function by multiple bioinformatics tools.

Mutations	SIFT	MutationTaster	PolyPhen-2
p.G439C	Damaging	Damaging	Damaging
p.C1616Y	Damaging	Damaging	Damaging

It is known that heterozygous mutations in the *COL4A3* gene are associated with benign forms of familiar proteinuria, which are usually neither progressive nor lead to renal failure.^[22]^ Looking at all the cases described previously, the first clinical manifestation of ARAS may appear as ephritic syndrome during pregnancy, as pregnancy-related hyperfiltration may acutely worsen renal function. Consistently, this study made it clear that ARAS should not be regarded as a benign condition for women during pregnancy. Currently, an effective anti-proteinuric therapy for ARAS, ACE inhibitors (ACE-I) and/or angiotensin receptor blockers (ARBs) are recommended.^[3,23]^ In our report, the 2 cases were treated with Losartan for anti-proteinuric therapy for 1 year, 24-h proteinuria decreased to pre-pregnancy level, suggesting the efficacy of Losartan is satisfactory.

## 4. Conclusion

This report highlights 2 cases of ARAS with progressive proteinuria but favorable pregnancy outcome, both of which enhance our understanding of *COL4A3* mutations-related ARAS. This study is intended to raise awareness of atypical courses of ARAS and emphasizes the importance of medical family history and genetic testing in such cases. Therefore, ARAS affected members and offsprings could receive treatments in time. Multidisciplinary treatments from nephrologists and gynecologists are needed to ensure the safety of pregnancy and the fetus. Further studies with a larger sample size of pregnancy in women with ARAS are required to analyze their pregnancy courses and outcomes in order to develop better evidence-based monitoring guidelines for ARAS.

## Author contributions

**Conceptualization:** Xiaoli Gao, Cha Han.

**Data curation:** Xiaoli Gao, Meilu Li.

**Investigation:** Meilu Li, Kan Wang, Zengyan Li.

**Supervision:** Xiaoli Gao.

**Writing – original draft:** Xiaoli Gao, Meilu Li.

**Writing – review & editing:** Cha Han.
